# Brain Gray Matter Atrophy after Spinal Cord Injury: A Voxel-Based Morphometry Study

**DOI:** 10.3389/fnhum.2017.00211

**Published:** 2017-04-28

**Authors:** Qian Chen, Weimin Zheng, Xin Chen, Lu Wan, Wen Qin, Zhigang Qi, Nan Chen, Kuncheng Li

**Affiliations:** ^1^Department of Radiology, Xuanwu Hospital, Capital Medical UniversityBeijing, China; ^2^Beijing Key Laboratory of Magnetic Resonance Imaging and Brain InformaticsBeijing, China; ^3^Department of Radiology, Dongfang Hospital Beijing University of Chinese MedicineBeijing, China; ^4^Department of Radiology, Tianjin Medical University General HospitalTianjin, China

**Keywords:** spinal cord injury, gray matter volume, voxel-based morphometry, brain atrophy, clinical variables

## Abstract

The aim of this study was to explore possible changes in whole brain gray matter volume (GMV) after spinal cord injury (SCI) using voxel-based morphometry (VBM), and to study their associations with the injury duration, severity, and clinical variables. In total, 21 patients with SCI (10 with complete and 11 with incomplete SCI) and 21 age- and sex-matched healthy controls (HCs) were recruited. The 3D high-resolution T1-weighted structural images of all subjects were obtained using a 3.0 Tesla MRI system. Disease duration and American Spinal Injury Association (ASIA) Scale scores were also obtained from each patient. Voxel-based morphometry analysis was carried out to investigate the differences in GMV between patients with SCI and HCs, and between the SCI sub-groups. Associations between GMV and clinical variables were also analyzed. Compared with HCs, patients with SCI showed significant GMV decrease in the dorsal anterior cingulate cortex, bilateral anterior insular cortex, bilateral orbital frontal cortex (OFC), and right superior temporal gyrus. No significant difference in GMV in these areas was found either between the complete and incomplete SCI sub-groups, or between the sub-acute (duration <1 year) and chronic (duration >1 year) sub-groups. Finally, the GMV of the right OFC was correlated with the clinical motor scores of left extremities in not only all SCI patients, but especially the CSCI subgroup. In the sub-acute subgroup, we found a significant positive correlation between the dACC GMV and the total clinical motor scores, and a significant negative correlation between right OFC GMV and the injury duration. These findings indicate that SCI can cause remote atrophy of brain gray matter, especially in the salient network. In general, the duration and severity of SCI may be not associated with the degree of brain atrophy in total SCI patients, but there may be associations between them in subgroups.

## Introduction

Previous studies on animals and humans have shown that brain cortex can be reshaped following spinal cord injury (SCI) (Pernet and Hepp-Reymond, 1975; Feringa and Vahlsing, 1985; Pons et al., 1991; Florence, 1998; Hains et al., 2003; Lee et al., 2004; Kim et al., 2006; Felix et al., 2012; Hou et al., 2014; Zheng et al., 2017). This cortical reorganization may contribute to the recovery of spared functions (Kaas et al., 2008; Ghosh et al., 2009; Kao et al., 2009), but it can also produce aberrant “sensations” without any external inputs, such as phantom sensations (Jain et al., 2000; Simoes et al., 2012) and neuropathic pain (Flor et al., 1995; Wrigley et al., 2009b; Henderson et al., 2011). To date, the mechanisms of cortical reorganization remain poorly understood, and most related research has been carried out on animals. Studies in humans require non-invasive approaches that were not available until relatively recently.

Currently, it remains uncertain whether these reorganizations result from new dendritic sprouting, or reflect an unmasking of already existing dormant synapses, or both. Endo et al. (2007) reported that in adult rats, the cortical representation in response to spare forelimb stimulation was enlarged and invaded into the adjacent sensory-deprived hind limb territory in S1 as early as 3 days after complete transection of the mid-thoracic spinal cord. The immediate cortical reorganization observed after spinal cord injury probably results from disinhibition or unmasking of existing dormant synapse after deafferentation (Aguilar et al., 2010). However, the extent of intracortical arborization of single thalamocortical axons is no >600 microns, and the shifts in cortical organization caused by latent synapses could be very small (<2–3 mm) (Rausell and Jones, 1995). Pons et al. (1991) reported dramatic expansion of S1 in monkeys following unilateral cervical dorsal rhizotomies, with the face representation expanding by up to 14 mm, so this cortical reorganization cannot be simply explained by the unmasking of latent connections and overlap of thalamocortical projection. The results from Pons and other researchers (Jain et al., 2000) support the idea that cortex reorganization may be driven dominantly by new dendritic sprouting rather than by the unmasking of already existing dormant synapses. Some researchers also indicated that the cortical changes caused by spinal cord injury were the sum of the two effects (Chen et al., 2002).

There have been a few non-invasive structural studies on brain gray matter (GM) change following SCI in humans, but these results were divergent (Crawley et al., 2004; Jurkiewicz et al., 2006; Wrigley et al., 2009a; Freund et al., 2011). For example, some researchers reported no changes in brain gray matter volume (GMV) in the primary motor cortex (Crawley et al., 2004; Jurkiewicz et al., 2006) after SCI, whereas others found a significant decrease in GMV in the same area in patients with SCI (Wrigley et al., 2009a; Freund et al., 2011). The wide range of disease duration, rehabilitation training, drug intervention, and different research methodology may have contributed to the inconsistencies between previous studies. Moreover, it is still unknown whether the anatomical changes occur only within the brain sensorimotor system, and furthermore, if there are any changes in brain GM following SCI, it is unclear whether these changes are correlated with injury severity, duration or clinical variables.

In this study, using a voxel-based morphometry (VBM) method (Ashburner and Friston, 2000), we aimed to explore the possible GMV changes voxel by voxel in whole brain regions after SCI and tried to elucidate the aforementioned issues. Based on previous studies (Hou et al., 2014), we hypothesized that brain GM impairment following SCI would be found in the primary sensorimotor cortex, and that longer and more severe injury would cause more significant changes in the GMV of the brain.

## Materials and methods

### Participants

In this study, 21 right-handed patients with SCI (15 male and 6 female patients, with a mean age of 50.5 ± 12.1 years, and an age range from 28 to 71 years) were enrolled. Lesions included cervical (eight cases), thoracic (one case), lumbar (five cases), and sacral (seven cases) levels. Impairment was labeled as grade A in 10 patients, and grade B, C or D in the remaining 11 patients, according to the American Spinal Injury Association (ASIA) Impairment Scale 2012 (http://asia-spinalinjury.org). The duration of the disease was from 1 month to 33 years, with a mean of 5.2 ± 8.7 years. The SCI group was divided into complete SCI (CSCI) group (10 cases) and incomplete SCI (ISCI) group (11 cases) based on the injury severity, and into sub-acute (duration 1–12 months; 8 cases) and chronic (duration >12 months; 13 cases) group based on the injury duration (Macklin et al., 2016). All patients had various degrees of bilateral sensorimotor dysfunction, with the exception of two patients who exhibited only unilateral dysfunction (one on the left and the other on the right side). All the patients had imaging hyper-intensity at the injury level on conventional T2-weighted MRI. We excluded the following patients from this study: patients with brain diseases that were confirmed by conventional MRI and patients with any type of mental illness or cognitive disorder. All patients underwent a comprehensive clinical assessment before MRI, including light touch sensory score, pinprick sensory score, and motor score, which were assessed by two qualified clinicians using the ASIA classification scale (Marino et al., 2003, 2008). A visual analog scale (VAS) and the Beck Depression Inventory (BDI) were also administered to evaluate the severity of pain and depression at the time of MRI acquisition. Sensory levels were assessed by testing two aspects of sensation: light touch and pinprick (sharp/dull discrimination) of each dermatome (C4–S5, bilateral). Motor function assessment comprised key muscle functions testing of 10 paired myotomes (C5–T1 and L2–S1; Jutzeler et al., 2016). The healthy control (HC) group comprised 21 right-handed healthy volunteers (15 men and 6 women, with a mean age of 50.0 ± 12.4 years and an age range of 26–65 years), matched for age, sex, and years of education. Table 1 provides detailed information of the patients with SCI.

**Table 1:** **Clinical data for the individuals with spinal cord injury**.

**ID**	**Age (years)**	**Sex**	**Etiology of the injury**	**Time since injury (years)**	Level of lesion^*^	**Side of the injury**	**ASIA score^†^**	**Motor (0–100)**	**Sensory^‡^ (0–224)**	**VAS**
1	55	F	Stab wound	0.75	C3–4	Left	D	80	113	6
2	50	M	Hit by weight	1	C6–8	bilateral	A	24	80	9
3	34	F	Vehicle accident	1	Sacral	Bilateral	D	74	190	4
4	38	M	Hit by weight	0.08	Lumbar	Bilateral	A	50	157	5
5	28	F	Fall injury	0.58	Sacral	Bilateral	D	70	160	0
6	51	M	Vehicle accident	1.33	Sacral	Bilateral	A	50	84	10
7	55	M	Hit by weight	9	Sacral	Bilateral	A	50	144	9
8	42	M	Hit by weight	9	Lumbar	Bilateral	A	56	160	9
9	38	M	Hit by weight	7	Lumbar	Bilateral	A	56	144	9
10	40	F	Injury by conveyor	12	Sacral	Bilateral	D	86	148	8
11	66	F	Stab wound	0.17	T10	Bilateral	C	80	172	0
12	52	M	Stab wound	0.25	Lumbar	Bilateral	A	50	168	0
13	60	M	Vehicle accident	3	C3–8	Right	C	70	204	9
14	33	M	Fall injury	0.1	Sacral	Bilateral	B	62	224	0
15	56	M	Injury by collapse	33	C4	Bilateral	A	60	158	8
16	51	M	Fall injury	0.08	C3–4	Bilateral	D	92	220	0
17	57	M	Stab wound	7	C4	Bilateral	D	90	152	6
18	65	F	Vehicle accident	0.17	C4–6	Bilateral	D	80	224	0
19	71	M	Fall injury	1+	C3–4	Bilateral	D	80	204	6
20	67	M	Vehicle accident	3	Lumbar	Bilateral	A	50	188	6
21	52	M	Hit by weight	11	Sacral	Bilateral	A	50	152	6

* The level of lesion refers to the neurological level.

† ASIA impairment scale: A: complete—no sensory or motor function is preserved in sacral segments S4–S5; B, incomplete—sensory but not motor function is preserved below the neurological level and extends through sacral segments S4−S5; C: incomplete—motor function is preserved below the neurological level, and more than half of the key muscles below the neurological level have a muscle grade of <3; D: incomplete—motor function is preserved below the neurological level, and at least half of the key muscles below the neurological level have a muscle grade of >3.

‡ *Sensory score: sum of segmental light touch and pinprick classifications. ASIA, American Spinal Injury Association. VAS, visual analog scale*.

The study protocol was approved by the Ethics Committee of Xuanwu Hospital of Capital Medical University, Beijing, China. Written informed consent in accordance with the Declaration of Helsinki was obtained from each participant of this study.

### Image acquisition

Images were obtained using a 3.0 T MRI system (Trio Tim, Siemens, Erlangen, Germany) with a 12-channel phase-array head coil. Tight but comfortable foam padding was used to minimize head motion, and earplugs were used to reduce the imaging noise. The participants were asked to stay calm, close their eyes, breath evenly, and try to avoid specific thoughts. Conventional brain axial fluid-attenuated inverse recovery sequence was used to exclude brain visible abnormality. High-resolution three-dimensional (3D) structural T1-weighted images were acquired in sagittal orientation using a 3D magnetization-prepared rapid gradient-echo sequence (MP-RAGE) with the following parameters: repetition time (TR) = 1800 ms; echo time (TE) = 2.13 ms; inversion time (TI) = 1100 ms; flip angle (FA) = 9°; number of slices = 192; slice thickness = 1 mm; field of view (FOV) = 256 × 256 mm^2^; matrix = 256 × 256; resulting an isotropic voxel size of 1 × 1 × 1 mm^3^.

### Data processing and VBM analyses

Post-processing was performed using Statistical Parametric Mapping (SPM8) software (http://www.fil.ion.ucl.ac.uk/spm/) implemented in MATLAB 2013a (Math Works, Natick, MA, USA), and included the following steps. All images were visually checked by two experienced radiologists to eliminate images with apparent artifacts caused by factors such as head motion, susceptibility artifacts, and instrument malfunction. All structural magnetic resonance images were then manually reoriented to place the anterior commissure at the origin and the anterior–posterior commissure in the horizontal plane. Next, the structural images were segmented into GM, white matter (WM), and cerebrospinal fluid (CSF) areas, using the unified standard segmentation option in SPM version 8. After segmentation, the individual WM and GM components were normalized into the standard Montreal Neurological Institute (MNI) space using the Diffeomorphic Anatomical Registration through Exponentiated Lie algebra (DARTEL) algorithm (Ashburner, 2007). The normalized GM component was modulated to generate the relative GMV by multiplied by the nonlinear part of the deformation field at the DARTEL step. The resulting GMV images were then smoothed with an 8-mm full-width at half-maximum Gaussian kernel.

### Statistical analysis

Both *T*-test and Partial correlation was used depend on the data with normal distribution, if not, rank sum test and spearman correlation test would be employed (Supplementary Material Tables S1, S2).

First, *T*-test was performed with age and sex between the SCI patients and HCs, and between the CSCI and ISCI sub-groups, and between the sub-acute and chronic sub-groups, no statistical significant differences were found (*p* > 0.05). Using the general linear model in SPM, a voxel-wise two-sample *t*-test was used to compare the GMV differences between the SCI group and the HC group (voxel-level uncorrected *p* < 0.0001, non-stationary cluster-level family-wise error correction with *p* < 0.05), with the age and sex as nuisance covariates. Next, the mean GMV values of clusters with statistical significance were extracted for subsequent *post-hoc* analyses. Then a region of interest (ROI)-based two-sample *t*-test was carried out to compare the GMV differences between the CSCI and ISCI sub-groups, and between the sub-acute and chronic sub-groups after removing age and sex effects (*p* < 0.05, uncorrected). Finally, partial correlation analysis was performed to explore any potential association between GMV and injury duration and clinical variables in patients with SCI, and between the GMV of each subgroup (CSCI and ISCI) and the duration of injury, and between the GMV and the clinical variables after removing age and sex effects (*p* < 0.05, uncorrected). The last several steps were also carried out in SPSS version 16 (IBM, Armonk, NY, USA).

## Results

### Brain GMV changes in patients with SCI

**Figure 1:**
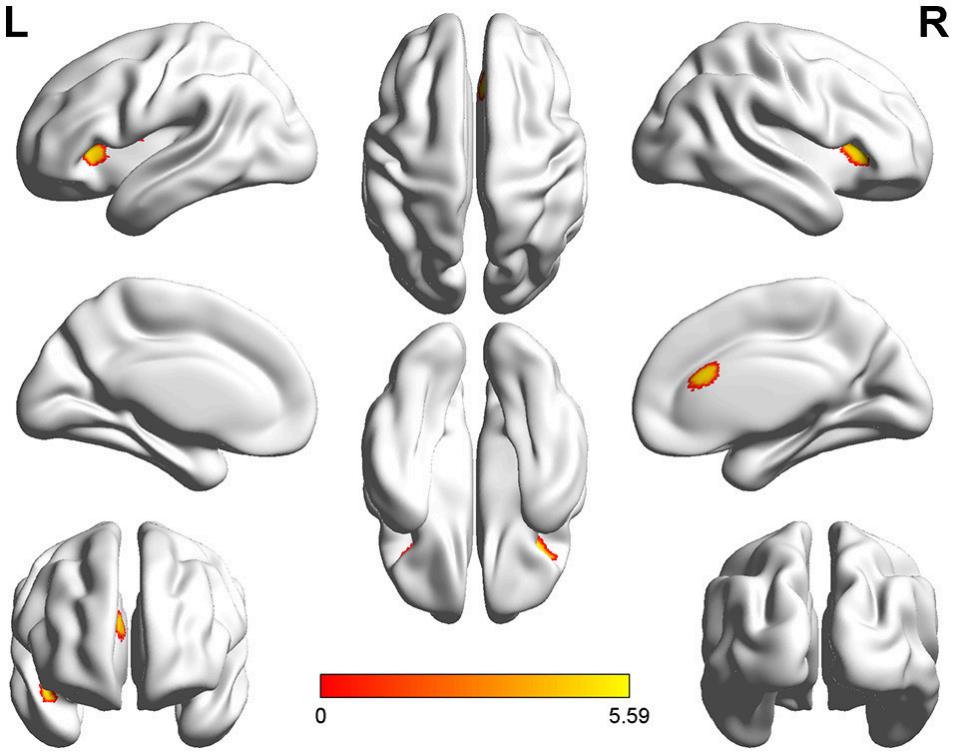
**Three-dimensional images made by BrainNet Viewer based on the result of VBM**. Regions showing significant higher GMV in healthy controls compared with the patients with SCI [cluster level, family-wise error (FWE) *p* ≤ 0.05]: dorsal anterior cingulate cortex (dACC), bilateral anterior insular cortex (aIC), bilateral orbital frontal cortex (OFC), and right superior temporal gyrus (STG).

**Figure 2:**
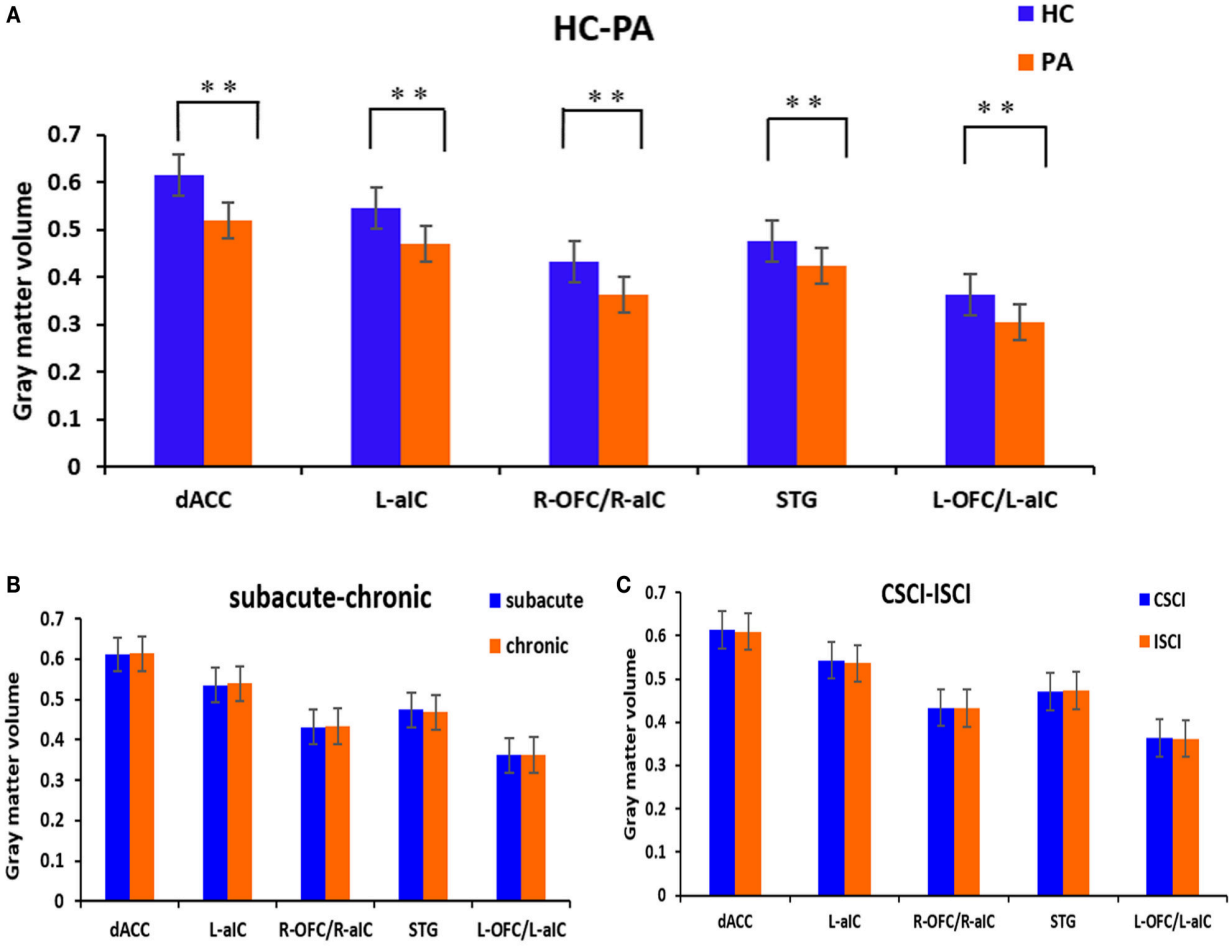
**GM volumetric changes between different sub-groups based on region of interest (ROI) analysis. (A)** Differences between the patients with SCI and healthy controls; **(B)** differences between the sub-acute and chronic sub-groups; **(C)** differences between the CSCI and ISCI sub-groups. ROIs were extracted based on the VBM findings. ^**^Statistical significance with cluster wise FWE-corrected *p* ≤ 0.05. dACC, dorsal anterior cingulate cortex; L-aIC, left anterior insular cortex; R-aIC, right anterior insular cortex; L-OFC, left orbital frontal cortex; R-OFC, right orbital frontal cortex; STG, right superior temporal gyrus.

Voxel-wise comparisons demonstrated that compared with HCs, patients with SCI had lower GMV in the dorsal anterior cingulate cortex (dACC), bilateral orbital frontal cortex (OFC), bilateral anterior insular cortex (aIC), and right superior temporal gyrus (STG) (non-stationary cluster-level FWE correction with *p* < 0.05; Table 2; Figures 1, 2A). There were no brain regions demonstrating higher GMV in the patients with SCI relative to the HCs (Figure 1).

**Table 2:** **Regions showing significant atrophy of gray matter volume in patients with SCI**.

**Regions with decreased GMV**	**Peak MNI coordinates**			**Cluster size (voxels)**	**Peak *T*-value**
	**X**	**Y**	**Z**		
ROFC/RaIC	36	27	6	518	5.44
LOFC/LaIC	−35	29	6	369	4.90
LaIC	−45	−6	3	236	5.14
Right superior temporal gyrus	44	−1	−17	403	5.10
Dorsal anterior cingulate cortex	1.5	24	24	326	5.59

*LaIC, left anterior insular cortex; LOFC, left orbital frontal cortex; RaIC, right anterior insular cortex; ROFC, Right orbital frontal cortex*.

### Differences in GMV between SCI sub-groups

ROI-wise comparisons revealed that there was no statistical difference in GMV between the sub-acute and chronic sub-groups (Figure 2B) or between the ISCI and CSCI sub-groups (Figure 2C) for the brain areas mentioned above (*p* > 0.05, uncorrected).

### Correlation between clinical variables and GMV in patients with SCI and between clinical variables and GMV of SCI sub-groups

**Figure 3:**
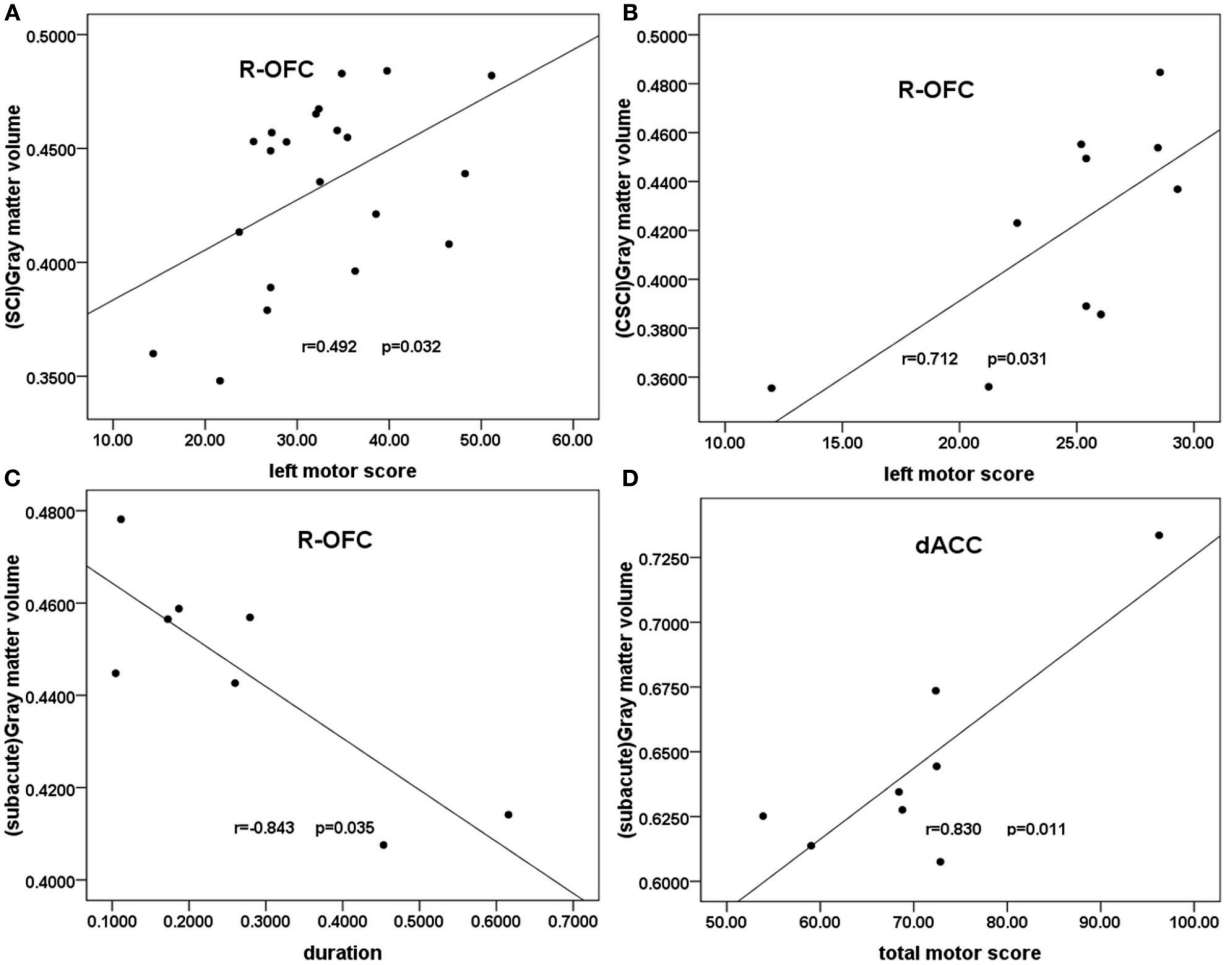
**Correlation between GMV and clinical scores in patients with SCI and the SCI subgroups**. Partial correlation showed **(A)** a moderate positive correlation between the GMV of the right OFC and the clinical left motor scores in patients with SCI (*r* = 0.492, *p* = 0.032); **(B)** a significant positive correlation between the right OFC GMV and the clinical left motor scores in the CSCI subgroup (*r* = 0.712, *p* = 0.031); **(C)** a significant positive correlation between the dACC GMV and the total clinical motor scores in the sub-acute sub-group (*r* = 0.830, *p* = 0.011); **(D)** a significant negative correlation between right OFC GMV and the injury duration in the sub-acute subgroup (*r* = −0.843, *p* = 0.035). R-OFG, right orbital frontal cortex; dACC, dorsal anterior cingulate cortex; GMV, gray matter volume; SCI, spinal cord injury.

In all patients with SCI, partial correlation analyses showed a trend toward negative correlation between right OFC GMV and the injury duration (*r* = −0.448, *p* = 0.055, uncorrected). A weak positive correlation was shown between the right OFC GMV and the clinical motor scores of left extremities (*r* = 0.492, *p* = 0.032, uncorrected; Figure 3A). In the CSCI subgroup, partial correlation analyses showed a significant positive correlation between the right OFC GMV and the clinical motor scores of left extremities(*r* = 0.712, *p* = 0.031, uncorrected; Figure 3B). In the sub-acute subgroup, a significant positive correlation was shown between the dACC GMV and the total clinical motor scores (*r* = 0.830, *p* = 0.011, uncorrected; Figure 3C), in addition, partial correlation analyses showed a significant negative correlation between right OFC GMV and the injury duration (*r* = 0.843, *p* = 0.035, uncorrected; Figure 3D). In the chronic subgroup, a trend toward positive correlation was found between the right OFC GMV and the clinical motor scores of left extremities (*r* = 0.599, *p* = 0.052, uncorrected).There were no statistical correlations between GMV and either VAS or BDI.

## Discussion

In the present study, we found significantly reduced GMV in many cognitive brain regions of patients with SCI compared with HCs. There were no statistical differences in GMV between the CSCI and ISCI sub-groups, or between the sub-acute and the chronic sub-groups. Finally, we found positive association between the GMV of patients with SCI and left motor scores, and between the GMV of CSCI patients and left motor scores. Additionally, in the sub-acute subgroup, a significant positive correlation was shown between the dACC GMV and the total motor scores, and a significant negative correlation was found between right OFC GMV and the injury duration. These findings may provide new insights into the structural plasticity of brain after SCI.

Brain GMV changes following SCI have been reported previously (Crawley et al., 2004; Jurkiewicz et al., 2006; Wrigley et al., 2009a; Freund et al., 2011, 2012, 2013; Felix et al., 2012), most of which were in the primary motor cortex (M1), primary somatosensory cortex (S1) and secondary somatosensory cortex (S2). Some researchers have suggested that these changes may be caused by direct or secondary Wallerian degeneration. As Freund et al. (2013) found that the corticospinal tracts and GM in the sensorimotor cortex of brain atrophied dramatically at 40 days after SCI, they proposed that volumetric changes in the brain GM and WM may be due to demyelination of axons and atrophy of neuronal cell bodies (Buss et al., 2004; Ramu et al., 2008; Cohen-Adad et al., 2011). In the present study, no volumetric changes were found in S1, M1, or S2, which is consistent with a few previous studies (Crawley et al., 2004; Yoon et al., 2013). An early study found that Wallerian degeneration of ascending fiber pathways following SCI can cause neuronal apoptosis, but the affected upward distance was only about 6–7 mm (Pallini et al., 1988). Thus, this short range of degeneration cannot involve the sensorimotor cortex, which could explain our findings.

In addition to direct degeneration of the sensorimotor cortex, SCI can also lead to atrophy of the non-sensorimotor cortex, such as the anterior cingulate cortex, insular cortex, and middle frontal gyrus (Wrigley et al., 2009a; Yoon et al., 2013; Jutzeler et al., 2016). For example, Wrigley et al. (2009a) have reported that patients with SCI had significant GM atrophy in the medial prefrontal cortex (MPFC) and anterior cingulate cortex. Consistent with their findings, we also found significantly decreased GMV in the dACC and bilateral anterior insular cortex, which are the core hubs of the salient network (Seeley et al., 2007). The anterior insula is one of the core hubs of the salient network that serves to identify the most relevant salient stimuli and forward them to higher cognitive regions to guide behavior (Menon and Uddin, 2010), while the dACC mainly plays roles in conflict monitoring between competing stimuli and provides a continuously updated prediction of expected cognitive demand to optimize future behavioral responses (Sheth et al., 2012). For patients with SCI, their sensory inputs were partially or fully lost. Thus, the reduced GMV in the core hubs of the salient network may reflect a disuse-induced transneuronal degeneration (Eidelberg et al., 1989; Bose et al., 2005; Jurkiewicz et al., 2006). We also found decreased GMV in the bilateral OFC. The OFC is an important relay of limbic system, and it is not only involved in regulating feeling and the visceral activities, but also in generating reward activity and some other complex psychological activity (Rolls, 2004; Cauda et al., 2011). Therefore, the decrease of GMV in OFC in patients with SCI may be associated with depression and with emotional and cognitive impairment after injury.

Although some earlier studies reported brain GM changes in patients with CSCI or patients with ISCI (Henderson et al., 2011; Villiger et al., 2015) they did not compare the potential differences directly between the two groups in one study. Thus, it is still uncertain whether injury severity can affect the level of GMV change. In this study, we directly compared brain GMV changes between patients with CSCI and patients with ISCI, but we found no statistical differences between them. Possible interpretations for this result are that: (1) the degeneration caused by SCI may be very slow and non-specific to both ISCI and CSCI; and (2) differences of the degree of brain atrophy between the CSCI and ISCI may be slight; and (3) the sample size of each SCI sub-groups was too small to identify tiny differences in GMV between the CSCI and ISCI.

The effect of SCI duration on cortical reorganization is not very clear. To date, only a few studies have explored the correlation between GMV and disease duration. Using an animal model, Rao et al. (2015) found positive correlations between regional homogeneity (ReHo) changes in the left ACC and the postoperative time. Several studies have shown that brain structure or function can be influenced by intervention during the duration of extensive disease following SCI (Corbetta et al., 2002; Cramer et al., 2007; Jurkiewicz et al., 2007; Jung et al., 2011; Lundell et al., 2011). However, there few studies have directly explored the correlation between injury duration and brain GMV changes. In the present study, we found a negative trend for the correlation between right OFC GMV of patients with SCI and injury duration (*p* = 0.055) and a significant negative correlation between right OFC GMV of sub-acute subgroup and injury duration (*p* = 0.035), indicating that longer disease course may lead to severer GM atrophy, especially in the sub-acute stage (duration 1–12 months), which is consistent with the findings of some previous studies (Freund et al., 2011, 2013). Of course, the reliability of this finding may benefit from larger sample sizes in future studies.

To date, there have been few studies exploring the correlation between GM changes and clinical variables. Hou et al. (2014) found a significant correlation between M1 GMV and total ASIA motor scores in patients with SCI. Villiger et al. (2015) reported a positive correlation between volumetric changes in the left middle temporal gyrus and the improvements in performance of patients with ISCI in rehabilitative training. In the present study, we found that right OFC GMV was positively related to left motor score not only in all the patients with SCI but also in the CSCI subgroup, moreover, a trend toward positive correlation was found between the right OFC GMV and the left motor scores in the chronic subgroup. Currently, a direct correlation between the OFC and motor function is lacking. It may contribute to the motor function indirectly, such as regulating reward activity (Rolls, 2004). Because the correlation is somewhat weak, we cannot make a clear inference. Additionally, we found a significant positive correlation between the dACC GMV and the total motor scores in the sub-acute subgroup(*p* = 0.011), indicating that the greater GMV in the dACC, the better clinical performance, which is consistent with the findings of some previous researches that: dACC play an important role in the brain work underlying motor control through its undirected and directed interactions with the supplementary motor area (SMA; Asemi et al., 2015; Diwadkar et al., 2017).

Several limitations of the present study should be addressed. First, we investigated GMV changes in patients with SCI with a very broad range of disease durations (range 1 month to 33 years). Second, the injured spinal segments were heterogeneous. Third, the severity of pain made the explanation more complex; however, we did not find a significant correlation between the GMV and the severity of pain. Finally, the relatively small sample size diminished the statistical power, especially when we tried to divide the patients with SCI into sub-groups.

In conclusion, our study provides evidence that SCI can cause significant atrophy of brain GM. Furthermore, brain atrophy was not evident in the sensorimotor system that directly innervates the paralyzed limbs, but in brain areas without direct connections, especially in the salient network. There were no statistical differences in GMV between the CSCI and ISCI sub-groups or between the sub-acute and the chronic sub-groups. But the GMV of the right OFC was correlated with the clinical motor scores of left extremities in not only all SCI patients, but especially the CSCI subgroup. In the sub-acute subgroup, a significant positive correlation was shown between the dACC GMV and the total clinical motor scores, and a significant negative correlation between right OFC GMV and the injury duration, indicating that the duration and severity of SCI may not be associated with the degree of brain atrophy in total SCI patients, but there may be associations between them in subgroups, which is need further study in future.

## Author contributions

QC and WZ: conception/design of the work; the acquisition, analysis, and the interpretation of data for the work; drafting of the work; final approval of the version to be published; and agreement to be accountable for all aspects of the work. XC/LW/WQ/ZQ: responsible for: the analysis data for the work; drafting of the work; final approval of the version to be published; and agreement to be accountable for all aspects of the work. NC: design of the work; revision of the work; final approval of the version to be published; and agreement to be accountable for all aspects of the work. KL: revising the work; final approval of the version to be published; and agreement to be accountable for all aspects of the work.

### Conflict of interest statement

The authors declare that the research was conducted in the absence of any commercial or financial relationships that could be construed as a potential conflict of interest.

## Supplementary material

Figure S1 **Brain white matter atrophy after spinal cord injury**.

## Abbreviations

GM: gray matter
GMV: gray matter volume
WM: white matter
CSF: cerebrospinal fluid
HC: healthy control
SCI: spinal cord injury
CSCI: complete spinal cord injury
ISCI: incomplete spinal cord injury
VBM: voxel-based morphometry
OFC: orbital frontal cortex
aIC: anterior insular cortex
STG: superior temporal gyrus
dACC: dorsal anterior cingulate cortex
SMA: supplementary motor area
TR: repetition time
TE: echo time TI, inversion time
FA: flip angle
FOV: field of view
ReHo: regional homogeneity
MNI: Montreal Neurological Institute.
