# Supplementary figures and images for: Brain Gray Matter Atrophy after Spinal Cord Injury: A Voxel-Based Morphometry Study
Source: Front Hum Neurosci. 2017 Apr 28;11:211. doi: 10.3389/fnhum.2017.00211 (PMC5408078; doi:10.3389/fnhum.2017.00211)

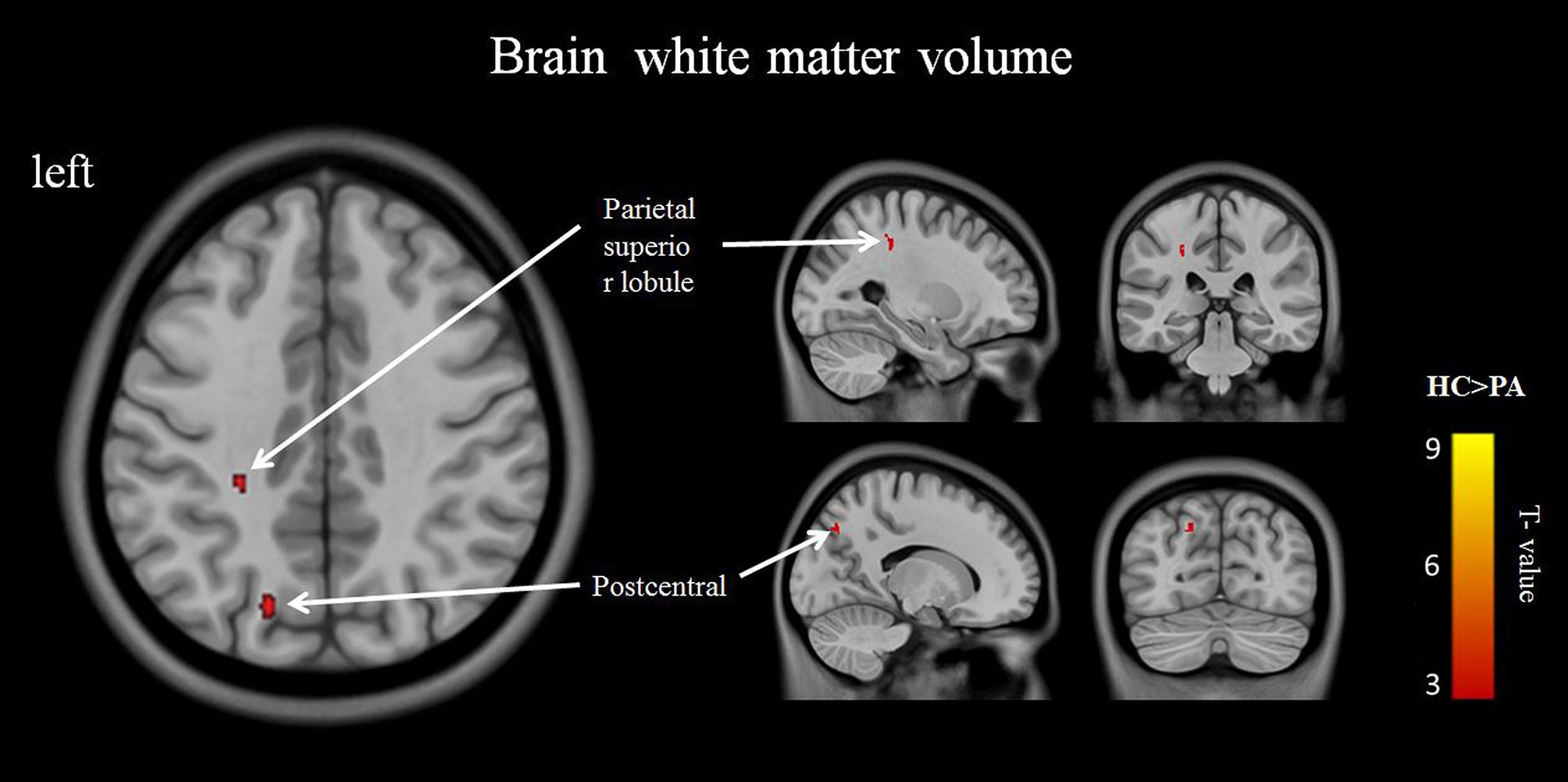

Supplement: Figure S1 — Brain white matter atrophy after spinal cord injury. [file Image1.TIF]
