# Supplementary material for: Brain Gray Matter Atrophy after Spinal Cord Injury: A Voxel-Based Morphometry Study
Source: Front Hum Neurosci. 2017 Apr 28;11:211. doi: 10.3389/fnhum.2017.00211 (PMC5408078; doi:10.3389/fnhum.2017.00211)
Supplement: Supplementary file 2 [file Table1.DOC]

TABLE S1 | Data showing normal distribution.
Tests of Normality	
	group	Shapiro-Wilk		
			Statistic	df	Sig.	
ROFC/RaIC	PA		.949	21	.320	
	NC		.966	21	.654	
LOFC/LaIC	PA		.931	21	.146	
	NC		.945	21	.278	
LaIC	PA		.936	21	.182	
	NC		.938	21	.199	
Right superior temporal gyrus	PA		.938	21	.195	
	NC		.936	21	.180	
Dorsal anterior cingulate cortex	PA		.924	21	.104	
	NC		.947	21	.297	
a. Lilliefors Significance Correction	
	
