# Supplementary material for: Brain Gray Matter Atrophy after Spinal Cord Injury: A Voxel-Based Morphometry Study
Source: Front Hum Neurosci. 2017 Apr 28;11:211. doi: 10.3389/fnhum.2017.00211 (PMC5408078; doi:10.3389/fnhum.2017.00211)
Supplement: Supplementary file 3 [file Table2.DOCX]

**Supplementary TABLE S2.**

1. **Results of partial correlations analysis between gray matter volume and clinic variables in SCI patients.**

| Regions with decreased GMV | Light touch# | | pinprick sensation* | | Sensory* | | motor* | | right motor* | | left motor* | | VAS# | | Duration* | |
| --- | --- | --- | --- | --- | --- | --- | --- | --- | --- | --- | --- | --- | --- | --- | --- | --- |
|  | r | p | r | p | r | p | r | p | r | p | r | p | r | p | r | p |
| ROFC/RaIC | 0.344 | 0.149 | 0.338 | 0.157 | 0.391 | 0.098 | 0.267 | 0.27 | -0.034 | 0.89 | 0.492 | 0.032 | -0.133 | 0.587 | -0.448 | 0.055 |
| LOFC/LaIC | 0.047 | 0.850 | 0.123 | 0.616 | 0.089 | 0.716 | 0.139 | 0.569 | 0.062 | 0.801 | 0.166 | 0.497 | -0.063 | 0.798 | -0.115 | 0.639 |
| LaIC | 0.037 | 0.818 | 0.199 | 0.405 | 0.119 | 0.629 | 0.114 | 0.943 | 0.241 | 0.32 | -0.079 | 0.748 | -0.081 | 0.741 | 0.149 | 0.542 |
| Right superior temporal gyrus | 0.019 | 0.380 | 0.211 | 0.386 | 0.113 | 0.646 | 0.013 | 0.957 | -0.036 | 0.882 | 0.102 | 0.678 | -0.202 | 0.408 | -0.381 | 0.107 |
| Dorsal anterior cingulate cortex | 0.114 | 0.642 | 0.201 | 0.409 | 0.171 | 0.483 | 0.112 | 0.647 | 0.23 | 0.343 | -0.081 | 0.74 | -0.343 | 0.151 | 0.103 | 0.676 |

Note: Correlation coefficient: r; Significance (2-tailed): p.;* normal distribution and partial correlation test was employed; # non normal distribution and spearman correlation test was used.

1. **Results of partial correlations analysis between gray matter volume and clinic variables in** **CSCI subgroup**

| Regions with decreased GMV | Light touch* | | pinprick sensation* | | Sensory* | | Motor# | | right motor# | | left motor# | | VAS# | | Duration# | |
| --- | --- | --- | --- | --- | --- | --- | --- | --- | --- | --- | --- | --- | --- | --- | --- | --- |
|  | r | p | r | p | r | p | r | p | r | p | r | p | r | p |  |  |
| LaIC | 0.452 | 0.222 | 0.450 | 0.225 | 0.451 | 0.223 | 0.390 | 0.299 | 0.266 | 0.490 | 0.403 | 0.282 | -0.141 | 0.717 | 0.103 | 0.793 |
| ROFC/RaIC | 0.349 | 0.357 | 0.350 | 0.356 | 0.350 | 0.356 | 0.342 | 0.367 | -0.044 | 0.910 | 0.712 | 0.031 | 0.051 | 0.895 | -0.373 | 0.322 |
| LOFC/LaIC | 0.447 | 0.227 | 0.450 | 0.224 | 0.449 | 0.226 | 0.331 | 0.384 | 0.058 | 0.883 | 0.558 | 0.119 | 0.064 | 0.870 | -0.102 | 0.794 |
| Right superior temporal gyrus | 0.239 | 0.536 | 0.240 | 0.533 | 0.240 | 0.534 | 0.214 | 0.580 | -0.132 | 0.736 | 0.578 | 0.103 | -0.041 | 0.917 | -0.405 | 0.279 |
| Dorsal anterior cingulate cortex | 0.552 | 0.123 | 0.552 | 0.123 | 0.552 | 0.123 | 0.041 | 0.917 | 0.012 | 0.975 | 0.062 | 0.875 | -0.426 | 0.252 | 0.049 | 0.900 |

Note: Correlation coefficient: r; Significance (2-tailed): p.; * normal distribution and partial correlation test was employed; # non normal distribution and spearman correlation test was used.

**C. Results of partial correlations analysis between gray matter volume and clinic variables** **in ISCI subgroup**

| Regions with decreased GMV | Light touch* | | pinprick sensation# | | Sensory* | | Motor* | | right motor* | | left motor* | | VAS# | | Duration# | |
| --- | --- | --- | --- | --- | --- | --- | --- | --- | --- | --- | --- | --- | --- | --- | --- | --- |
|  | r | p | r | p | r | p | r | p | r | p | r | p | r | p | r | p |
| ROFC/RaIC | 0.009 | 0.918 | 0.333 | 0,381 | 0.299 | 0.435 | -0.577 | 0.104 | -0.565 | 0.113 | -0.109 | 0.780 | -0.251 | 0.516 | -0.330 | 0.386 |
| LOFC/LaIC | -0.241 | 0.532 | -0.168 | 0.665 | -0.256 | 0.506 | -0.081 | 0.836 | 0.029 | 0.941 | -0.158 | 0.684 | -0.178 | 0.647 | -0.034 | 0.930 |
| LaIC | 0.036 | 0.927 | -0.311 | 0.415 | -0.259 | 0.501 | 0.302 | 0.430 | 0.430 | 0.247 | -0.388 | 0.302 | 0.077 | 0.844 | 0.018 | 0.963 |
| Right superior temporal gyrus | 0.158 | 0.684 | -0.168 | 0.666 | -0.078 | 0.841 | -0.261 | 0.498 | -0.064 | 0.870 | -0.375 | 0.319 | -0.365 | 0.334 | -0.457 | 0.217 |
| Dorsal anterior cingulate cortex | 0.101 | 0.796 | -0.024 | 0.951 | 0.023 | 0.952 | 0.511 | 0.160 | 0.526 | 0.146 | 0.068 | 0.862 | -0.355 | 0.349 | 0.035 | 0.928 |

Note: Correlation coefficient: r; Significance (2-tailed): p. * normal distribution and partial correlation test was employed; # non normal distribution and spearman correlation test was used.

**D. Results of partial correlations analysis between gray matter volume and clinic variables in sub-acute subgroup**

| Regions with decreased GMV | Light touch# | | pinprick sensation# | | Sensory* | | Motor* | | right motor* | | left motor* | | VAS# | | Duration# | |
| --- | --- | --- | --- | --- | --- | --- | --- | --- | --- | --- | --- | --- | --- | --- | --- | --- |
|  | r | p | r | p | r | p | r | p | r | p | r | p | R | p | r | p |
| ROFC/RaIC | -0.090 | 0.865 | 0.577 | 0.231 | 0.485 | 0.330 | 0.321 | 0.535 | 0.020 | 0.970 | 0.564 | 0.244 | -0.274 | 0.600 | -0.834 | 0.035 |
| LOFC/LaIC | -0.401 | 0.430 | 0.077 | 0.885 | -0.095 | 0.858 | 0.434 | 0.390 | 0.266 | 0.610 | 0.510 | 0.302 | -0.112 | 0.832 | -0.212 | 0.687 |
| LaIC | 0.291 | 0.576 | -0.441 | 0.381 | -0.280 | 0.591 | 0.569 | 0.239 | 0.729 | 0.100 | 0.266 | 0.610 | 0.216 | 0.681 | 0.378 | 0.460 |
| Right superior temporal gyrus | 0.115 | 0.828 | 0.030 | 0.956 | 0.074 | 0.889 | 0.795 | 0.059 | 0.696 | 0.125 | 0.713 | 0.112 | -0.256 | 0.625 | 0.094 | 0.859 |
| Dorsal anterior cingulate cortex | 0.177 | 0.738 | 0.091 | 0.864 | 0.155 | 0.770 | 0.830 | 0.011 | 0.713 | 0.112 | 0.760 | 0.079 | -0.218 | 0.678 | 0.146 | 0.782 |

Note: Correlation coefficient: r; Significance (2-tailed): p. * normal distribution and partial correlation test was employed; # non normal distribution and spearman correlation test was used.

**E. Results of partial correlations analysis between gray matter volume and clinic variables** **in chronic subgroup**

| Regions with decreased GMV | Light touch* | | pinprick sensation* | | Sensory* | | Motor* | | right motor* | | left motor* | | VAS* | | Duration# | |
| --- | --- | --- | --- | --- | --- | --- | --- | --- | --- | --- | --- | --- | --- | --- | --- | --- |
|  | r | p | r | p | r | p | r | p | r | p | r | p | r | p | r | p |
| ROFC/RaIC | 0.511 | 0.108 | 0.511 | 0.108 | 0.511 | 0.108 | 0.242 | 0.473 | -0.066 | 0.847 | 0.599 | 0.052 | -0.005 | 0.988 | -0.487 | 0.129 |
| LOFC/LaIC | 0.325 | 0.330 | 0.325 | 0.330 | 0.325 | 0.330 | 0.214 | 0.528 | 0.073 | 0.832 | 0.303 | 0.365 | 0.102 | 0.764 | -0.085 | 0.804 |
| LaIC | 0.112 | 0.744 | 0.112 | 0.744 | 0.112 | 0.744 | -0.041 | 0.906 | 0.074 | 0.891 | -0.314 | 0.347 | -0.343 | 0.301 | 0.214 | 0.527 |
| Right superior temporal gyrus | 0.246 | 0.466 | 0.246 | 0.466 | 0.246 | 0.466 | -0.046 | 0.894 | -0.239 | 0.479 | 0.194 | 0.568 | 0.011 | 0.975 | -0.410 | 0.211 |
| Dorsal anterior cingulate cortex | -0.075 | 0.827 | -0.075 | 0.827 | -0.075 | 0.827 | -0.214 | 0.527 | -0.074 | 0.830 | -0.414 | 0.206 | -0.057 | 0.867 | 0.349 | 0.292 |

Note: Correlation coefficient: r; Significance (2-tailed): p.; * normal distribution and partial correlation test was employed; # non normal distribution and spearman correlation test was used.
