# Supplementary material for: Brain Gray Matter Atrophy after Spinal Cord Injury: A Voxel-Based Morphometry Study
Source: Front Hum Neurosci. 2017 Apr 28;11:211. doi: 10.3389/fnhum.2017.00211 (PMC5408078; doi:10.3389/fnhum.2017.00211)
Supplement: Supplementary file 4 [file Table3.DOCX]

**Supplementary STABLE 3 | Regions showing significantly atrophy of white matter volume in SCI patients**

| WM decreased regions | Peak MNI coordinates | | | Cluster size  (voxels) | Peak *T* value |
| --- | --- | --- | --- | --- | --- |
|  | X | Y | Z |  |  |
| Parietal superior lobule | -18 | -75 | 42 | 36 | 4.85 |
| Postcentral gyrus | -28.5 | -36 | 43.5 | 32 | 4.29 |

Regions showing significant lower white matter volume in the SCI patients compared with healthy controls

based on voxel-based morphometry(VBM) (cluster *P*<0.001, topoFDR *P*<0.05 ). It is overlaid on the

MNI152_T1_1mm template. They are as follows: the parietal superior lobule, postcentral gyrus.
